# PAIN ASSESSMENT AND MANAGEMENT IN NURSING HOME RESIDENTS WITH ADRD: INSIGHTS FROM INTERDISCIPLINARY TEXT MESSAGES

**DOI:** 10.1093/geroni/igae098.4040

**Published:** 2024-12-31

**Authors:** Ashley Woods, Matthew Farmer, Mihail Popescu, Kimberly Powell

**Affiliations:** University of Missouri, Santa Fe, New Mexico, United States; University of Misosuri, Colombia, Missouri, United States; University of MIssouri, University of Missouri, Missouri, United States; University of Missouri, Columbia, Columbia, Missouri, United States

## Abstract

The study aims to evaluate the relationship between the representation of pain assessment and management in interdisciplinary text messages about older adults residing in Nursing Homes (NH) with and without Alzheimer’s Disease and Related Dementias (ADRD) before NH to hospital transfer. A retrospective cohort study and secondary data analysis of (n=21,000) text messages exchanged among healthcare team members caring for NH residents who transferred to the hospital from 2016 to 2020 was performed. Natural language processing was used to identify mentions of pain assessment, management, and pain medications in text messages. A two-sample independent T-test was used to test our hypothesis. A negative binomial generalized linear model was used to assess the association between pain mentions and various resident characteristics, including ADRD status. The analysis included 694 transfer events involving 414 residents, 39% of whom had ADRD. Significant differences were found in the t-test with pain assessment and management mentions, with ADRD residents having fewer mentions (t= 2.38, p=0.02) of pain assessment and management than those without ADRD. The generalized linear model identified statistically significant predictors (age, race, and specific comorbidities) of the number of text messages exchanged among NH residents, but interestingly, dementia stage and avoidable transfers were not significant. Our findings indicate that pain is underrepresented in communications regarding ADRD residents, as well as Black patients, potentially contributing to disparities in pain assessment and management in these populations.

